# Cool Communities—Urban Density, Trees, and Health

**DOI:** 10.3390/ijerph15071547

**Published:** 2018-07-22

**Authors:** Helen Brown, Katrina Proust, Barry Newell, Jeffery Spickett, Tony Capon, Lisa Bartholomew

**Affiliations:** 1 WHO Collaborating Centre for Environmental Health Impact Assessment, School of Public Health, Curtin University, Perth, Western Australia 6102, Australia; j.spickett@curtin.edu.au (J.S.); larkinlisaj@gmail.com (L.B.); 2 Fenner School of Environment and Society, Australian National University, Canberra, ACT 0200, Australia; Katrina.proust@anu.edu.au (K.P.); barry.newell@anu.edu.au (B.N.); 3 School of Public Health, The University of Sydney University, Sydney, NSW 2006, Australia; tony.capon@sydney.edu.au

**Keywords:** trees, cities, health, climate change, Urban Heat Island (UHI)

## Abstract

A move towards more compact and climate-resilient cities is being encouraged around the world. As part of these plans, there is a need to manage the potential conflict between increasing urban densities and the extent of tree canopy in cities. Reductions in tree canopy are a major contributor to the urban heat island (UHI) effect, which will act to reduce rather than increase climate resilience in many cities. A systems thinking approach called Collaborative Conceptual Modelling was used to study the interaction between urban infill, tree canopy, and human health in Perth, Australia. The results indicated that under current planning policies and development practices, the behaviour of the system is dominated by the drive towards higher housing densities. While this may result in the attainment of urban infill targets, it is likely to lead to a reduction in tree canopy, higher temperatures, and a decrease in a range of other benefits provided by trees. Recommended actions to overcome this behaviour were determined by the identification of leverage points in the system. These included a shift to a sustainable development paradigm that places greater value on the environmental and social benefits provided by trees and a greater emphasis on a climate-resilient future. Market and legislative mechanisms should be integrated into the city’s greening strategy and development plans to ensure the protection of existing trees and the inclusion of new trees on public and private land.

## 1. Introduction

Strategies that encourage the development of climate-resilient cities should be an integral part of climate change adaptation. Climate change projections for higher temperatures can be compounded in cities by the creation of urban heat islands (UHI). The UHI effect is caused by the typical characteristics of urban areas that result in higher temperatures than their surrounding non-urban areas [1,2,3]. These include lower rates of evapotranspiration due to less vegetation, greater storage of heat from solar radiation due to lower surface albedo, and more anthropogenic sources of heat [2,4]. Numerous cities around the world have reported UHI effects ranging from 2 °C to 12 °C, depending on the individual characteristics of a city [3,5,6,7,8,9]. A study of 50 of the most populous cities in the United States (US) reported that between 1961 and 2010, temperature increased at twice the rate of background global increases for all of the cities that had undergone substantial urbanization during that period [10]. Another study in China reported an average increase in average air temperatures due to urbanization of 0.124 degrees for every decade [4]. UHI can also be intensified by heatwaves [11], which are likely to occur with higher frequency and duration as a consequence of climate change [12]. These and other studies demonstrate the potential of the UHI effect to amplify the warming expected from climate change.

The potential public health implications of higher temperatures, particularly heatwaves, are well documented. The European heatwave of 2003 recorded approximately 45,000 deaths [13]. In Australia, heatwaves have resulted in more fatalities than all other natural hazards combined [14]. Studies reporting heat-related mortality has been reported in all of the capital cities in Australia [15]. A recent example is the estimated 500 excess deaths recorded in the 2009 heatwave in southeast Australia [16]. Heat-related deaths are often linked to the exacerbation of existing conditions such as cardiovascular disease or respiratory illness [14,17,18,19]. Health impacts can also be compounded by increases in ground level ozone, which can occur as a result of higher temperatures [20,21]. Vulnerability to the health effects of heatwaves or extreme heat can be affected by exposure to heat, sensitivity to that exposure, and the capacity to adapt by implementing suitable coping strategies.

The combined effect of climate change and UHI should be considered in light of increasing urbanization. The percentage of the world’s population living in urban areas has increased from 30% in 1950 to a current level of 55% and a projected level of 68% by the year 2050 [22]. Given these increases and the well-established deleterious effects of extreme heat on human health, it is critical that the potential influence of urban planning on ambient temperatures is prioritized.

This paper reports on a case-study in Perth, Western Australia (WA) that focuses on the interaction between the built environment and heat in the context of climate change adaptation. Perth is an isolated urban centre undergoing significant population growth and ageing. The number of heat-related deaths in Perth is expected to double by the year 2050 [23]. A previous study, which used a Health Impact Assessment (HIA) methodology, determined that the projected increase in heatwaves and days over 35 °C presented one of the highest risks to human health associated with climate change in WA [24,25,26]. The study also recognized that exposure to heat could be exacerbated in some areas of Perth due to the occurrence of the urban heat island (UHI) effect.

A fundamental policy response of Perth’s projected population increase has been urban consolidation, primarily through urban infill in inner metropolitan areas. This significant change in urban form has the potential to contribute to the formation of UHIs over the coming decades. Mitigation strategies for the UHI effect include increasing urban vegetation, increasing the albedo of built surfaces and reducing waste heat emissions. Reductions of between 1 °C and 7 °C in city-wide air temperatures have been reported as a result of UHI mitigation [10]. Vegetative strategies, particularly tree planting, are generally found to be one of the most effective of all of the UHI mitigation strategies [27,28,29].

A study in Perth was conducted as part of the national Commonwealth Scientific and Industrial Research (CSIRO) Urbanism, Climate Adaptation, and Health Research Cluster (the Cluster). The study combined HIA and systems approaches to develop effective adaptive strategies to reduce the potential health impacts of climate change in urban areas in Australia. The study identified heatwaves as the greatest health risk related to climate change in Perth. Following this, systems approaches were used to consider strategies to reduce health impacts associated with heatwaves and higher temperatures. This paper reports on this phase of the research. The study received ethics approval from the Human Research Ethics Committee at Curtin University.

Systems approaches support understanding and decision-making in complex systems. Interactions in complex systems typically include a network of circular feedback effects, so as one variable in the system is changed, a range of outcomes, both intended and unintended, will occur [30,31]. A common response to complexity is to divide an issue into segments and design policies within that segment; however, policies that emerge from this process are often ineffective in the long run because they are not based on an understanding of the behaviour of the wider system [32,33]. A range of different methods can be employed when undertaking a systems approach, many of which involve model building in group situations and cycles of elicitation, integration, representation, and reflection [34,35,36,37,38,39,40,41]. This type of collaborative modelling process is particularly useful when different experiences and viewpoints of the challenge can improve understandings of the structure and behaviour of the system [34]. In a systematic review of the use of systems thinking in public health research, Carey et al. [39] reviewed methods ranging from qualitative, action-based research to quantitative dynamic model building, concluding that the use of qualitative techniques were likely to be the most useful approach for public health research. 

## 2. Methods

The method used in this study was collaborative conceptual modelling (CCM), which is a systems approach developed by Newell and Proust that has been explained elsewhere in greater detail [36,42]. This approach captures different viewpoints to create a shared understanding of the issue of interest, culminating in ‘dynamic hypotheses’ that describe simple feedback structures with the potential to dominate system behaviour. This understanding can be used as a basis for decision-making within the issue of interest.

CCM consists of a series of six co-evolving activities that can be tailored to suit the characteristics and objectives of the particular research. The activities conducted in this project were:

• What is the Challenge?

**Figure 1:**
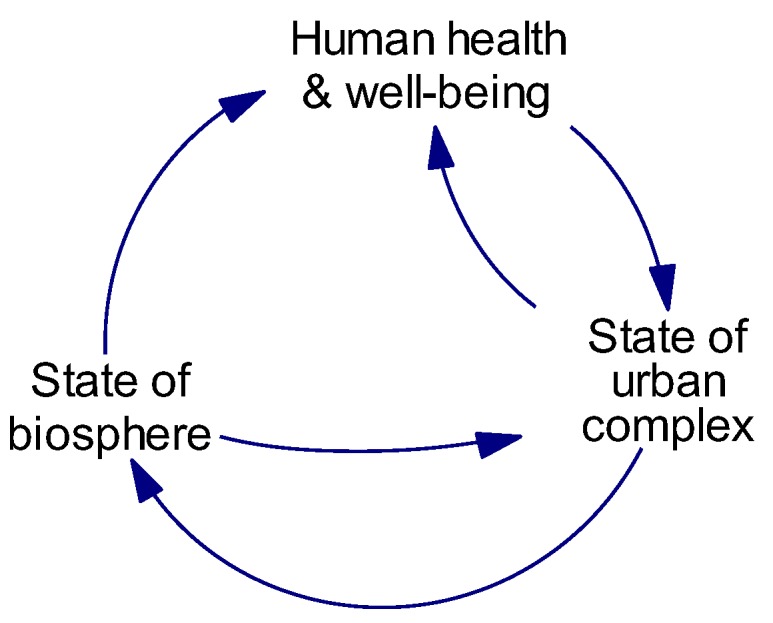
The Co-Effects Template.

The step defines the key challenge faced in a particular situation and requires the input of a range of stakeholders. This activity was guided by a pre-existing conceptual model that was developed as part of the broader cluster and previously described by Proust el al [42]. The model, called the ‘Co-Effects Template’ (Figure 1), was developed after several individual project workshops, and a final integrative workshop with nine participants from projects cutting across topics of thermal stress, urban food systems, and climate change adaptation. The template captures a high-level view of the urban climate–health system that can be applied to multiple challenges.

• What is the Story?

The second step considered the effect of past and current policies on trees in urban Perth. An existing review of the impact of historical planning policy and legislation on trees in Perth provided an assessment of previous policies. Content analysis of current planning policy documents was undertaken using a coding scheme based on key words related to vegetation, climate, and health. Similar approaches have been used in a textual analysis of the extent to which health is incorporated into the planning strategies of major Australian cities [43]. The policies reviewed were: Directions 2031; State Planning Policies of Urban Growth and Settlement and Residential Design Codes; Liveable Neighbourhoods; Capital City Planning Framework; and Climate Change Management Draft Guidelines (WA Local Government Association). A review of the State Emergency Response Plan for Heatwaves (WESTPLAN Heatwave) and the State Planning Policy for Natural Hazards and Disaster was also undertaken as part of this activity.

• Can I see how you think?

This CCM activity reflects the need to share different perspectives (or mental models) of a particular issue to enhance understanding of the system, and was conducted as a half-day workshop. Key stakeholders with a clear connection to the issue were identified, including the state government (Departments of Health, Planning, Environment and Conservation and Water, the Environmental Protection Authority and the Building Commission), local government, private developers, academics, and professional institutes. A total of 32 representatives from the health, planning, development, and environment sectors across state and local government and the private sector attended the workshop. The key focus of the workshop was the level of tree canopy in areas targeted for urban infill. Each participant was provided with basic instructions on how to construct a simple model called an influence diagram. Influence diagrams are a simple qualitative systems model designed to capture the key interactions, including the feedback structure, of the topic of interest, and have been used to investigate health issues such as obesity, safety, and healthy eating habits [38,44,45].

Participants were given 15 minutes to draw an individual influence diagram depicting their viewpoint of the issue, including: three to four key variables that have the largest influence on tree canopy levels in Perth; three to four of the most important effects of tree canopy; and feedback links between these variables. Each participant was then allocated a partner from a different sector and asked to explain their influence diagram. Each pair had 15 minutes to construct a ‘pair-blended’ diagram that integrated both viewpoints. The diagrams were presented and discussed at table groups, and were also displayed around the room for participants to review. The importance of how those involved in collaborative modelling articulate their own views and then develop a common understanding has been highlighted by Oppl [41].

Participants were then divided into six groups and used the pair-blended influence diagrams to guide the selection of eight key variables influencing tree canopy management in urban infill areas. The involvement of people with relevant knowledge and expertise to construct models is likely to produce models that are more useful for informing work processes [46,47]. A simple scoring system was utilized with each participant provided with three coloured stickers worth one, two, or three points that were assigned to the three most important variables. The scores were combined and the variables were ranked. Each group then discussed one of the top six variables. The discussion was guided by key categories of: opportunities, barriers, potential conflict or synergies with other activities or sectors, and the identification of key stakeholder groups. 

• What drives system behaviour?

Information from the policy review and workshop was analysed to identify key feedback structures and consider which elements of the system currently have the greatest level of influence over tree canopy levels. This informed the development of additional models that depict key system characteristics such as feedback loops and delays that typically drive system behaviour. As well as updated influence diagrams, other typical qualitative systems models such as stock-and-flow maps and system archetypes were developed [30,32,37,42,48].

• What are the leverage points?

Leverage points are places within a complex system where a small shift in one thing can produce significant and enduring changes in the system. Identification of these points was facilitated by a consideration of Meadows’ [49] classification, which includes the mindset and goals of the system, the rules and who controls them, information and materials flows, feedback loops, and numbers such as subsidies and taxes. System archetypes, which are generic feedback structures with characteristic behaviour that occur in a wide range of situations, were also used in this step. An understanding of these structures and their behaviour can facilitate the identification of effective management strategies [30,32,34,50].

• Can we have new eyes?

The final activity of CCM reconsiders the original challenge defined in the first activity. The understanding gained from the CCM process supports new mental models or ‘new eyes’ that can help develop more effective management strategies to the challenge.

## 3. Results

Results are presented under the key headings of the first five CCM steps.

### 3.1. What Is the Challenge?

**Figure 2:**
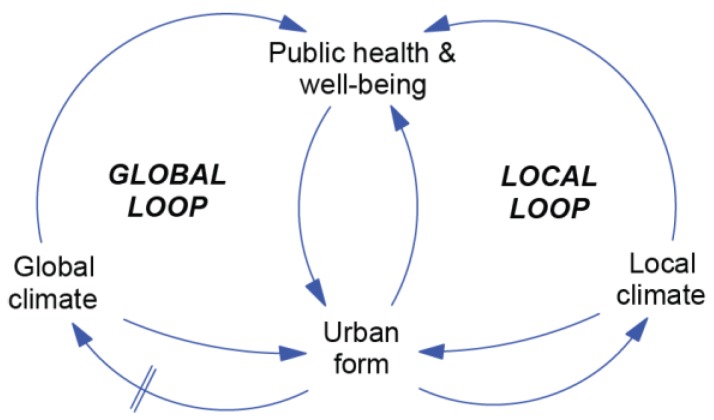
Global–Local Model.

In the first activity, ‘What is the Challenge?’, a variation of the Co-Effects Template was developed focusing on the selected challenges of temperature, urban form, and public health. The resulting conceptual model was named the Global–Local Model (Figure 2), and highlights that the interaction between urban areas, temperature, and health occurs at the global and local scale.

The two loops of the model reflect the considerable differences in the spatial and temporal scales of the interactions between urban form and climate. The ‘Global Loop’ is relatively unresponsive to changes in local urban form due to the small fraction of global greenhouse gas (GHG) emissions from Perth, and the inertia of the climate system. This inertia results in a delay between GHG emissions and a change to the climate represented by the parallel lines on the link between ‘Urban form’ and ‘Global climate’. In contrast, the influence of the UHI effect in the ‘Local Loop’ is more immediate and determined by the natural and built characteristics of Perth. In terms of exposure to heat, strategies targeting the Local Loop represent the best opportunity at the local level to progress towards a more climate-resilient city. 

**Figure 3:**
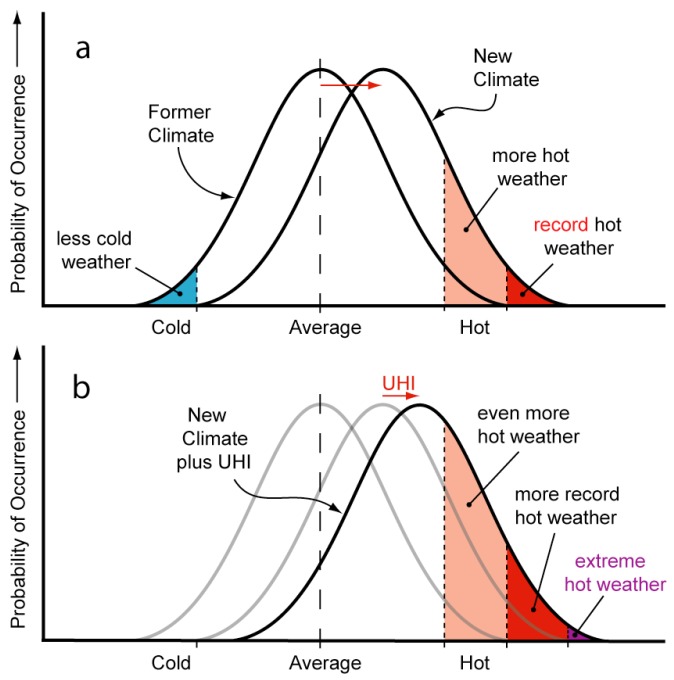
The Combined Impact of Climate Change (**a**) and the Urban Heat Island (UHI) Effect (**b**).

The relationship between urban form and local climate within the context of climate change is depicted in Figure 3. The diagram shows how a shift in the distribution of climatic conditions affects the frequency of extreme temperatures. Panel (a) depicts the increased probability of hot weather and record hot weather as a result of climate change [51,52], and panel (b) overlays a second shift that can occur in urban areas as a result of the UHI effect.

The second shift in temperature distribution will present multiple challenges. The challenge selected for this study was the management of tree canopy in areas targeted for urban infill and higher densities.

### 3.2. What Is the Story?

The second activity considered the historical review of the impact of planning policy and legislation on trees in Perth by Brunner and Cozens [53]. This review indicated that the regulation and protection of trees in urban areas had been given limited attention in planning policy. More recent reductions in vegetation became apparent around the year 2000, and coincided with a change in the type of infill development. The authors concluded that “the protection of mature vegetation and trees in WA has been afforded little time or consequence”.

A content analysis of key planning policy documents highlighted climate aspects that focused on climate change mitigation, particularly the need for increased energy efficiency and the transport efficiencies of compact cities. Although ambient temperature is a critical determinant of energy use, the effect of urbanization and tree canopy changes on ambient temperature was largely overlooked in the policies reviewed. The potential loss of tree canopy as a result of urban consolidation and the issue of UHIs are only mentioned in two non-statutory documents: the Capital City Planning Framework and the Climate Change Management Draft Guidelines. No explicit UHI mitigation requirements were included in the statutory planning policies that were reviewed.

The primary focus of the WESTPLAN Heatwave is managing health outcomes related to heatwaves. The plan discusses the increased risk of extreme heat associated with climate change and the UHI effect. It also recognizes the links between heatwaves and infrastructure such as roads, railways, bridges, power supply, and industry. In contrast, the Natural Hazards and Disaster Policy, which aims to plan for natural disasters as a fundamental element of all planning documents, makes no specific mention of heatwaves, referring only to floods, cyclones, storm surges, severe storms, landslides, bushfires, and earthquakes. This suggests that the connection between the built environment and extreme heat are not afforded a high priority in natural hazard planning in WA. This view is supported by the Natural Hazard Risk in Perth study, which stated that heatwaves are probably the most underrated weather hazard in Australia [54].

### 3.3. Can I See How You Think?

**Figure 4:**
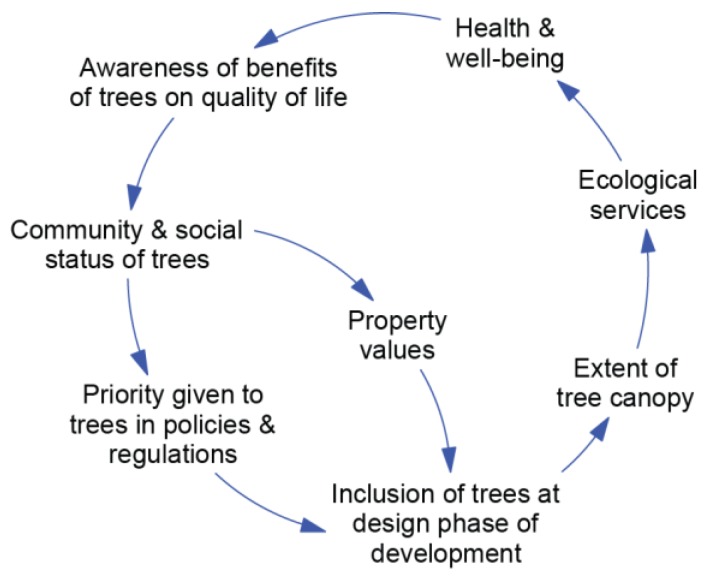
Health and Environment Sector Influence Diagram.

**Figure 5:**
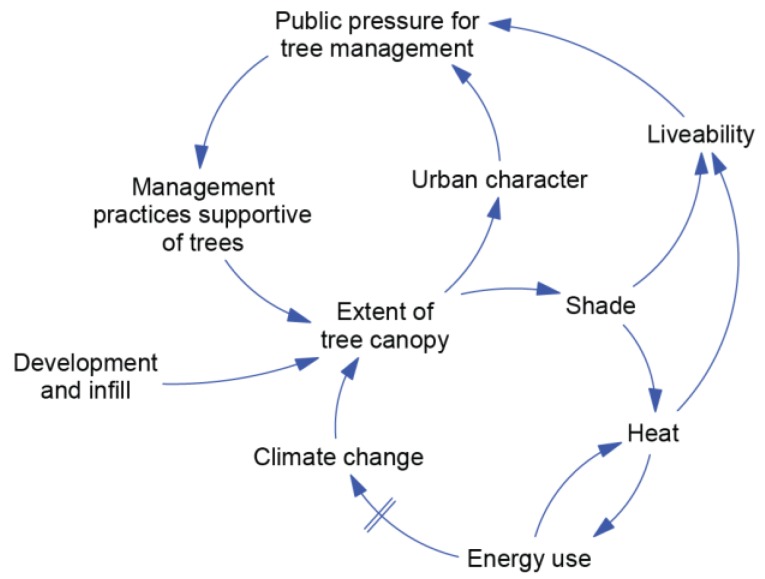
Planning and Environment Sector Influence Diagram.

**Figure 6:**
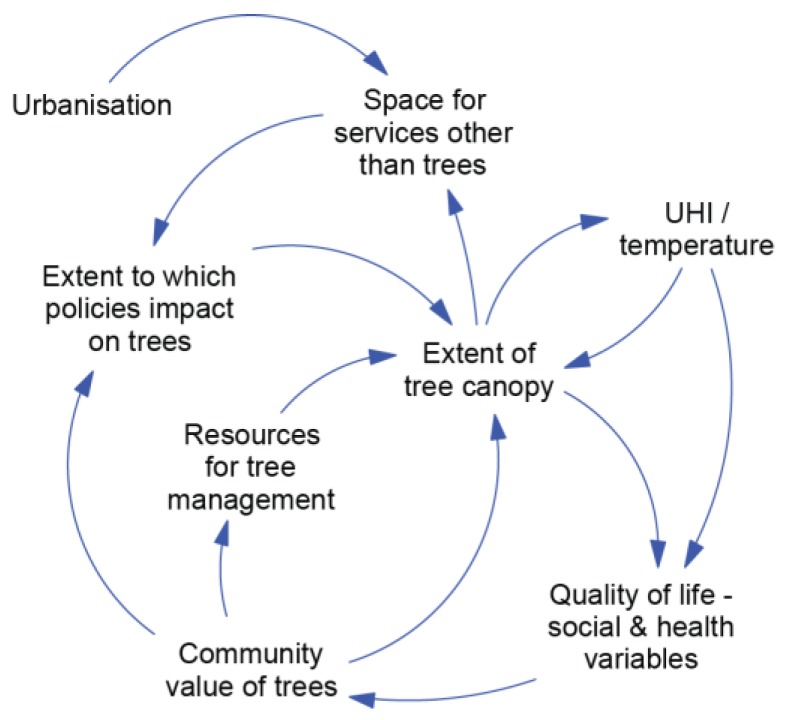
Development and Environment Sector Influence Diagram.

The systems workshop produced 16 pair-blended influence diagrams, as described in the methods. A selection of these diagrams is shown in Figure 4, Figure 5 and Figure 6, with each of these representing a dynamic hypothesis of the system.

The hypothesis represented in this influence diagram (Figure 4) is that the extent of tree canopy affects a range of ecological services (including shade, visual amenity, and reduction in heat and air pollutants) that has flow-on effects to health and well-being. As these effects are realized, this leads to greater awareness of the benefits of trees on quality of life, which affects the status given to trees by the community. This status can influence the extent to which trees are included in the design phase via both policy and market (property values) mechanisms.

Figure 5 depicts how management practices can be influenced by different factors over time. Urban infill and development has a direct influence on the extent of tree canopy. Tree canopy loss leads to reductions in shade and higher temperatures; both have detrimental effects on liveability, which is defined in the Capital City Framework as “the capacity of a place to deliver the everyday qualities of life to which most of us aspire”. This includes qualities such as accessibility to amenities, a safe and healthy living environment, a sense of community, vibrancy, and choice [55]. Urban character was selected by this pair as a separate variable. They also showed that increases in heat can escalate as higher energy use (air conditioning) releases more waste heat into the environment. This section of the diagram represents important aspects of the UHI effect, and also recognises the impact on the Global Loop, as higher energy use results in increases in greenhouse gas emissions that contribute to climate change, which over time may influence the health and extent of tree canopy.

As these effects are realized, public pressure is exerted on management practices to counter the original reductions in tree canopy. The types of processes include members of the community lobbying local government or local politicians to retain trees in their community, the formation of community groups that increase awareness of the issue through direct involvement in the community, and consumer pressure for property in areas with good tree cover.

A shared viewpoint of participants from the development and environment sectors is shown in Figure 6. As urbanization increases, there are more space demands for services and infrastructure such as housing, roads, driveways, and utilities. This leads to competition for space. As the availability of space is reduced, policies and practices will affect tree canopy either directly (such as street tree policy) or indirectly (drainage, essential services). The extent of tree canopy will influence the available space for other services. This ‘loop’ is essentially about a number of variables that in some circumstances are viewed as ‘competing interests’. A related workshop comment was that the protection or provision of trees is given a lower priority than many other infrastructure requirements.

Another feedback loop is shown where the changes in tree canopy can lead to increases in temperature, which may influence tree health and canopy levels. The change in temperature and tree canopy levels affects a broad range of social and health variables that influence the overall quality of life. Once again, the participants saw the community value of trees as a key variable in influencing tree canopy via a combination of resources for tree management and changes in the extent to which other policies impact on trees.

Subsequent group discussions identified and ranked the most critical variables in determining tree canopy levels. The top six variables with total scores in brackets were:

- Urbanization policies, regulations, and practice (35)
- Community values and expectations (33)
- Health and well-being (28)
- Urban design (15)
- Market values and drivers (12)
- Economic value of trees (8)

The qualitative models presented at the workshop were influenced by the opinions and expertise of the workshop participants. Further research could test or expand these models with other key stakeholders.

### 3.4. What Drives System Behaviour?

The information from each of the previous steps was analysed post-workshop. Features considered critical to determining system behaviour, such as goals, rules, information flows, and feedback structures, were identified. Analysis of these features provided significant insight into the factors that have the most influence on tree canopy levels in areas of urban infill in Perth. These factors included:

- Planning policies and practices are driven predominantly by the goal of a more compact city, resulting in significant reductions in tree canopy.
- A lack of policies and regulations to support the inclusion and retention of trees in a more compact city.
- Accounting processes with regard to urban infrastructure do not adequately capture the value of services provided by trees or the cost of their removal.
- A lack of awareness about the range of benefits provided by tree canopy and the long-term costs associated with their removal.

**Figure 7:**
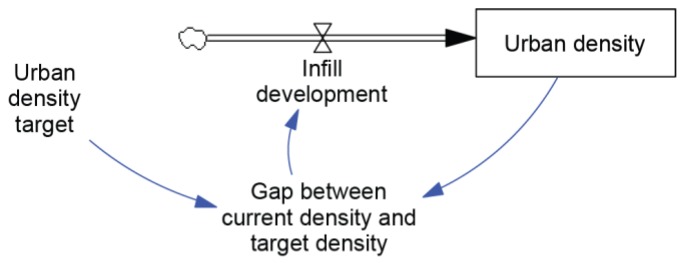
The Compact Mechanism.

Urban consolidation policies were seen as a strong influence on system behaviour. The vision of Directions 2031 is for “a world-class liveable city: green, vibrant, more compact, and accessible with a unique sense of place”. The “more compact” element of this vision is supported by a clear target of an extra 154,000 infill dwellings and the Urban Growth Monitor, which reports on the construction of dwellings in urban areas [56]. Figure 7 is a stock-and-flow map that depicts how these support mechanisms drive progress towards the infill target. The key stock (represented by the rectangle) is urban density, which is measured by the Urban Growth Monitor. The rate of infill development (represented by the tap symbol) is driven by the size of the gap between the current level of urban density and the urban-density target. As urban density approaches the target level, the gap between the two will reduce, the rate of infill development will slow down, and ideally, the urban density target will be met. These mechanisms are supported by a range of policies and market interest in meeting the demand for new dwellings.

**Figure 8:**
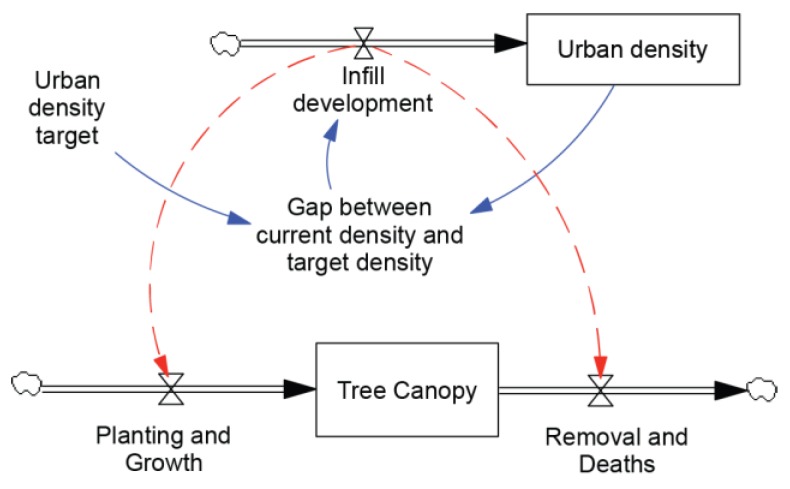
Consequences of Urban Infill on Tree Canopy Levels.

The influence that the ‘Compact Mechanism’ has on tree canopy stock is represented by the dotted links in Figure 8. Findings from the policy review and the workshop activities support the conclusion that current urban infill practices typically reduce opportunities to plant trees and increase the rate of removal of trees, both leading to a decline in tree canopy. Directions 2031 provides support for a more compact city, but contains no specific tree canopy targets, and no provision for monitoring tree canopy levels, planting, or the removal of trees. We conclude that, in the context of Directions 2031, a reduction of tree canopy levels in areas of urban infill are likely to be a side effect of mechanisms to support a more compact city.

**Figure 9:**
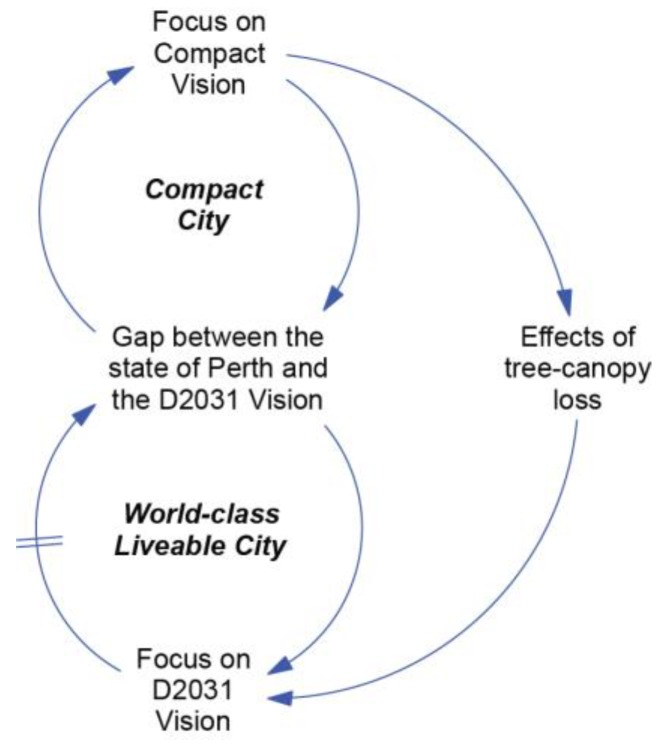
Tunnel Vision.

The relationship between urban density and tree canopy levels depicted above can be analysed using system archetypes. In Figure 9, the relationship between the Compact City and the World-Class Liveable City is expressed in terms of an archetype called “Shifting the Burden” [32]. This archetype depicts a situation where the gap between the desired state and the current state of the system can be managed by two alternative solutions: a symptomatic solution or a fundamental solution. Symptomatic solutions are frequently applied because they respond to a problem symptom and are often *part of* the fundamental solution; however, they can also undermine progress towards the desired outcome in other ways. If management of the system is focused primarily on the Compact Vision, other important aspects of liveability such as tree canopy can be neglected, which is expressed in Figure 9 as ‘Tunnel Vision’. In the long run, the adverse effects may be recognised, and result in a shift to the fundamental solution. However, in this situation, the shift is limited by factors that are difficult to reverse, such as the removal of mature canopy trees and urban design that fails to incorporate trees. 

The failure of the current system to account for the value of trees and the external costs associated with reductions in tree canopy was identified as a significant barrier to the effective management of tree canopy in urban infill areas. Although the ecosystem services provided by trees are critical to a healthy urban environment, the value of these services is largely absent from traditional economic approaches that drive budgets and private developers. While it was recognised that there was a willingness to pay a premium for greener areas that could support market mechanisms to increase tree canopy, it was considered likely to be limited to higher socio-economic areas. This is supported by studies in other cities that have shown that low income areas recorded lower rates of vegetation [57,58] as well as higher temperatures [58].

The extent of community awareness influences tree canopy via pressure on policy makers and the market place to incorporate trees into urban environments. The strength of this feedback is a critical determinant of the system’s behaviour; however, evidence from this study indicates that at the time of this study, this feedback loop is relatively weak. As stated in the Capital City Planning Framework [55], the incremental nature of tree canopy loss often means that it “goes unnoticed until it is too late to undertake preventative actions”. In effect, as levels gradually decline, lower tree canopy levels may become the new norm.

### 3.5. What Are the Leverage Points?

The most powerful leverage point in Meadow’s list [49] is paradigms. While paradigms are difficult to change, they have the potential to influence decision-making across all levels and aspects of the system. The paradigm shift required here is to move from a system dominated by narrow or short-term economic considerations to one that is based on a sustainable development paradigm that takes into account the environmental, social, and economic costs and benefits of tree canopy in urban areas for current and future generations.

Goals capture the purpose or function of the system, and are a powerful leverage point because they are an indication of what we value most and can have a significant influence on all aspects of the system [31]. While the study highlighted that there were clear goals for urban density targets, these were lacking for tree canopy. Introducing a specific goal or target for tree canopy levels would create a strong leverage point that could serve as an additional indicator of progress towards a more liveable city.

Effective goals must be supported by rules such as policies and laws, incentives, constraints, and disincentives. If the goal of the system has been narrowed, the rules will tend to follow suit. The review of past and current policies and the findings of the workshop indicated that current rules do not provide adequate protection of tree canopy levels in Perth. Rules pertaining to tree planting and removal, on both private and public land, are critical. One example highlighted in the workshop was the need for rules to reduce the widespread practice of removing all vegetation, particularly mature trees, during urban development. The physical limitations created by other policies, such as zoning limitations, setback, and height limits and requirements for other infrastructure may severely limit the physical opportunities to incorporate trees into the urban design. If current rules discourage the inclusion of trees in urban design, these rules should be reconsidered. Community tree planting programs can play a significant role in tree management plans, and have the added advantage of increasing community awareness regarding the importance of trees in urban environments. However, these are typically limited to public land, so the influence on the critical space of private land is limited.

Information flows are strong leverage points in changing system behaviour. The study indicated that there are significant gaps and biases in the information flows of the system. In the absence of thorough tree canopy monitoring and reporting, tree canopy levels will continue to be largely overlooked. Improvements in information flows are also needed with respect to the links between tree canopy levels and a range of social, environmental, and economic factors. Responsibility for these factors is dispersed across many sectors, and highlights the need for a whole-of-government approach to manage tree canopy. 

## 4. Discussion

The discussion is focused on the final activity of CCM, ‘Can we have new eyes?’ The shift to a more compact city is not unique to Perth. Increasing awareness of the high economic, environmental, and social costs of urban sprawl is creating a global move towards more compact cities [59]. The considerable benefits of compact cities include the more efficient delivery of essential infrastructure, goods, and services, and reduced environmental costs of land-clearing and long commute times. Compact cities also promote health, with ‘walkable’ distances encouraging active transport and physical activity in general [60]. Perth is one of the most sprawling cities in the world, and progress towards a more compact city, especially in light of the anticipated population growth, is clearly warranted. However, care must be taken so that the higher densities are planned and designed in a way that avoids undesirable consequences such as those associated with lower tree canopy levels.

The interest in this research stemmed from the relationship between urban form, tree canopy, and temperature, within the context of a warming climate in Perth, which is a city that regularly experiences temperatures in excess of 40 °C during summer. This research supports the findings of others that the management of climate impacts within urban areas is generally given only minor consideration within planning frameworks and development policy [5,61]. In a review of climate action plans from cities in the US, Stone, Vargo, and Habeeb [10] concluded that excluding UHI mitigation strategies from plans may “fail to adequately protect human health and welfare from rapidly rising temperatures”. The evidence from this study suggests that the UHI effect is not given significant consideration in planning frameworks, and the risk to human health in Perth is likely to increase without action. The inclusion of heatwaves in the Natural Hazards and Disaster Policy would be an appropriate starting point for considering the influence and role of the planning sector with regard to the urban climate. 

Subsequent to this research, the Perth and Peel@3.5million report was released in March 2018 [62]. The report extends strategic land-use planning beyond Direction 2031 to the year 2050. Some elements of the report that are relevant to this research are encouraging. The report states that “enhancing the urban forest and increasing tree canopy cover is the most efficient and economical way to reduce the effects of urban heat island in communities” (p72). The importance of this issue was also raised by the Environmental Protection Authority response to the draft report, which recommended actions to counteract the effects of the UHI effect through a greening strategy for the region [63]. Information flows have also improved with widespread mapping of the urban forest; however, these have raised further concerns. The Urban Forest of Perth and Peel Statistical Report stated that tree canopy levels in Perth are highly variable with some areas recording less than 5% coverage [64]. Other studies have reported similar levels of variability, with the lowest levels often recorded in areas of lower socio-economic status. The Urban Forest report also highlights that trees on residential land account for a significant proportion of the urban forest in many areas, and that these trees (and areas) are at greatest risk due to policies that support increases in density while offering limited protection for established trees. While the Perth Peel report updated urban infill targets to 377,130 new infill dwellings by 2050 and recognised the potential impact on the tree canopy levels of these dwellings, the report does not include a tree canopy target or a clear strategy on how the risk to tree canopy and flow-on effects will be addressed. Improvements in the availability of tree canopy and urban infill could be used to develop and test quantitative system dynamic models in future research.

Another recent report on Urban Forests from the Western Australian Local Government Association (WALGA) provides additional insights into the findings of this study [65]. A survey involving 33 Local Government Areas (LGAs) highlighted that many are actively addressing the challenge of falling tree canopy levels in urban areas with initiatives such as street tree policies and urban forest plans (78% and 60% of LGAs respectively). However, the key finding was that without changes to “state government planning legislation, policies, and plans … local government will struggle to maintain and grow their urban forests, particularly on private land and verges” (p6). In addition, plans for a framework to support local government management of the issue were considered to be “of no value unless there is clear head of power/legislation to give local governments the ability to enforce it” (p4).

These recent findings support the findings of the case study, which indicate that the behaviour of the current system is dominated by actions to increase urban density whilst failing to provide protection for urban canopy levels. The series of leverage points identified during the study provide a range of management options that can lead to a more sustainable approach where a balance between urban density and tree canopy is achieved.

## 5. Conclusions

In the context of increasing urbanization and climate change, a shift towards more climate-resilient cities should be at the forefront of urban planning. This fundamental shift is required for climate change adaptation across all sectors, and is related to many impacts, including human health. This study used a system thinking approach to consider how past and present policies and practices have influenced tree canopy levels in Perth. The study concluded that policies and practices that are intended to increase urban densities will continue to result in reductions in tree canopy levels unless several key changes are implemented. 

The leverage points discussed in this paper provide guidance in this respect: a shift to a sustainable development paradigm that places greater emphasis on the environmental and social aspects related to tree canopy; clear goals for tree canopy levels; strong policies and regulations to support these goals; the power to enforce these, particularly in high-risk areas such as private land; and greater consideration of the interaction between urban planning policies, climate, and human health. Delays in the implementation of these actions is likely to lead a more climate-sensitive city and increased risks to public health.
